# Liver Fibrosis in Primary Sjögren’s Syndrome

**DOI:** 10.3389/fimmu.2022.889021

**Published:** 2022-06-15

**Authors:** Theodoros Androutsakos, Theodoros A. Voulgaris, Athanasios-Dimitrios Bakasis, Maria-Loukia Koutsompina, Loukas Chatzis, Ourania D. Argyropoulou, Vasilis Pezoulas, Dimitrios I. Fotiadis, George Papatheodoridis, Athanasios G. Tzioufas, Andreas V. Goules

**Affiliations:** ^1^ Department of Pathophysiology, School of Medicine, National and Kapodistrian University of Athens, Athens, Greece; ^2^ Department of Gastroenterology, School of Medicine, National and Kapodistrian University of Athens, General Hospital of Athens “Laiko”, Athens, Greece; ^3^ Unit of Medical Technology and Intelligent Information Systems, University of Ioannina, Ioannina, Greece; ^4^ Biomedical Research Section, Institute of Molecular Biology and Biotechnology, Ioannina, Greece

**Keywords:** non-alcoholic fatty liver disease, Sjögren’s syndrome, liver fibrosis, liver steatosis, transient elastography

## Abstract

**Background:**

Primary Sjögren syndrome (pSS) is a systemic autoimmune epithelitis, potentially affecting salivary epithelium, biliary epithelium, and hepatocytes. Common immunological mechanisms might cause clinically silent liver inflammation, and combined with non-alcoholic fatty liver disease (NAFLD), liver fibrosis (LF) may occur. No studies have explored the occurrence of LF in the context of NAFLD among pSS patients.

**Methods:**

Consecutive pSS patients from the rheumatology outpatient clinic of the Department of Pathophysiology and individuals evaluated in the hepatology outpatient clinic for possible NAFLD serving as comparators underwent transient elastography (TE) to assess LF and liver steatosis (LS). All participants had no overt chronic liver disease. Clinical, demographic, and laboratory data were collected from all participants at the time of TE.

**Results:**

Fifty-two pSS patients and 198 comparators were included in the study. The median age (range) of pSS and comparators was 62.5 (30–81) and 55 (19–86) years, respectively. Both groups had similar prevalence regarding type 2 diabetes mellitus, hyperlipidemia, and similar body mass index (BMI). Patients with pSS had less frequently high LS (S2, S3) (27% vs. 62%, *p* < 0.001) and significant LF (F2–4) [2 (3.8%) vs. 34 (17.2%), *p* = 0.014] than comparators. Univariable analysis showed that advanced LF was significantly associated with older age, higher LS, greater BMI, and disease status (comparators than pSS); of these, only age was identified as an independent LF risk factor in the multivariable logistic regression analysis.

**Conclusion:**

Liver fibrosis among pSS patients is most likely not attributed to the disease *per se*.

## Introduction

Liver fibrosis (LF) is characterized by excessive deposition of fibrous tissue into hepatic parenchyma that may progress into cirrhosis and liver failure. The most common etiologies of LF include non-alcoholic fatty liver disease (NAFLD) and alcoholic fatty liver disease, viral infections, drugs, and other less common chronic liver diseases either metabolic or autoimmune such as hemochromatosis, Wilson’s disease, primary biliary cholangitis (PBC), and autoimmune hepatitis (AIH). More specifically, NAFLD comprises a major health problem worldwide with a rapidly rising incidence and a prevalence of approximately 25%–35% in the general population (1–4). The most common predisposing factors of NAFLD are obesity, metabolic syndrome, type 2 diabetes mellitus (T2DM) and hyperlipidemia, all of which display an increasing incidence (1, 5, 6). NAFLD is in fact an umbrella disease, including all manifestations of lipid accumulation in the liver, ranging from simple liver fat deposition in more than 5% of hepatocytes [non-alcoholic fatty liver (NAFL)] to liver necroinflammation [non-alcoholic steatohepatitis (NASH)], fibrosis, and cirrhosis (7, 8). Liver biopsy (LB) is still the gold standard for diagnosis and staging of liver fibrosis (LF) and liver steatosis (LS), but it is an expensive and invasive procedure with a high sampling error and a high risk of complications including pain, bleeding, and very rarely death (9–13). LF can be assessed by various non-invasive methods, with transient elastography (TE) being one of the most reliable when performed by an experienced operator (14–17). Similarly, the presence of fat in the liver parenchyma can be easily diagnosed by several imaging modalities including TE (9, 10).

On the other hand, primary Sjögren’s syndrome (pSS) is a systemic autoimmune disease characterized by chronic lymphocytic infiltration of exocrine glands and especially salivary and lachrymal glands, with the potential to affect almost any internal organ. Liver has been traditionally considered as one of the most commonly involved extra-glandular organ in the context of pSS, although the exact prevalence varies widely among different studies mainly due to the heterogeneity of the definition of hepatic disease. Sjögren’s syndrome-associated liver disease is clinically expressed as abnormal liver function tests, most likely due to PBC and/or AIH. Older studies supported that liver disease prevalence ranges between 5% and 27% (18–20), while more recent studies report even higher prevalence varying from 10% to 49% (21–23). Apart from pSS-associated PBC and AIH, LFT elevation among pSS patients could be attributed also to NAFLD, drug hepatotoxicity, and hepatitis B and C infections (21–27). Thus, the chronic and long-standing inflammatory state observed in the liver of pSS patients combined with other common comorbidities might promote the development of liver fibrosis in pSS sub-clinically and before overt liver involvement. Surprisingly, no epidemiologic or pathophysiological studies have been conducted to explore the association of LF and NAFLD in the context of pSS. The current study aims to investigate the prevalence of LF among pSS patients without overt liver disease, by performing TE, in comparison with NAFLD individuals, and after careful consideration of common comorbidities and potential confounders.

## Materials and Methods

### Study Design and Participant Population

Consecutive pSS patients aged >18 years, who fulfilled the 2016 ACR/EULAR criteria and were followed in the rheumatology outpatient clinic of the Department of Pathophysiology at the General Hospital of Athens “Laiko” from June 1 to December 31 of 2021, were included in the current study. Individuals who were evaluated in the hepatology outpatient clinics of the Department of Pathophysiology and the Department of Gastroenterology at the General Hospital of Athens “Laiko” for possible NAFLD based on ultrasonographic criteria (higher echogenicity than renal cortex) served as the comparator group.

The exclusion criteria were as follows: (i) patients who were concomitantly diagnosed with other systemic autoimmune rheumatic disease or IgG4-related disorder; (ii) patients with incomplete laboratory testing, such as antinuclear antibody, anti-SSA/Ro, and anti-SSB/La, at diagnosis; (iii) patients who did not undergo minor salivary gland biopsy; (iv) patients who were under chemotherapy for solid or hematological malignancies; (v) patients with transaminasemia at the time of TE; (vi) patients with either medical history or laboratory tests suggesting non-NAFLD chronic liver diseases including chronic hepatitis B and C infections, heart failure, alcoholic or metabolic liver diseases, drug hepatotoxicity, as well as the presence of liver cirrhosis; and (vii) patients demonstrating hepatic structural abnormalities on ultrasonography that may affect TE. Transaminasemia was defined as the presence of alanine (ALT) and/or aspartate (AST) aminotransferases >3× the upper limit of normal (ULN) and alkaline phosphatase >2× ULN, while cirrhosis was diagnosed based on previous TE, radiographic, biochemical, or histological findings according to guidelines (28).

All participants underwent TE by a specialized operator blindly, to measure both liver stiffness for LF and controlled attenuation parameter (CAP) for LS. For LF, the following liver stiffness cutoffs were used: F0–F1: 2–7 kilopascals (kPa), F2: 7–10 kPa, F3: 10–14 kPa, and F4: >14 kPa. The LF stage of F0–F1 was considered as clinically insignificant and F2–F4 was considered as significant (29). For LS, the following CAP cutoffs were used: S0: 100–238 decibels/meter (dB/m), S1: 238–260 dB/m, S2: 260–290 dB/m, and S3: >290 dB/m. LS stage S0–S1 was classified as low and S2–S3 was classified as high (29). Patients were characterized as having diabetes if they were on antidiabetic treatment or if their blood sugar level during testing was >126 mg/dl or >200 mg/dl for fasting or non-fasting patient, respectively, and as hyperlipidemic if they were on hypolipidemic treatment or if their fasting cholesterol level was >200 mg/dl and/or LDL was >130 mg/dl after double checking at least 3 months apart (30, 31). In order to exclude concomitant liver diseases other than NAFLD, laboratory testing for exclusion of viral and autoimmune hepatitis was performed before TE, while alcohol-related liver disease was defined as liver damage attributed to alcohol overuse (more than 3 drinks on any day or more than 7 drinks per week for women and more than 4 drinks on any day or more than 14 drinks per week for men) according to national abuse and alcoholism guidelines (32). Demographic, clinical, and laboratory data were recorded for all participants, while for pSS patients, the immunologic profile, the cumulative dose and duration of methotrexate and prednisone, and the European League Against Rheumatism Sjögren's Syndrome Disease Activity Index (ESSDAI) at the time of diagnosis were additionally calculated and collected (33, 34). All systemic manifestations of pSS were defined based on the ESSDAI definitions and small airways as described previously (34, 35). Patients with pSS (study group) were compared with individuals with NAFLD (comparator group) in terms of LF and LS, and various subgroup analyses were performed.

All tests were routinely performed as standard of care, while the TE procedure was non-invasive. Therefore, ethical committee approval was unnecessary. All patients were explicitly informed about the nature of the study and the investigations performed, and they gave written consent for their participation and use of data for research purposes. The study was in compliance with the general protection rule regulation (GDPR) of the European Union.

### Statistical Analysis

Statistical analysis for numerical data was performed with Mann–Whitney or *t*-test after applying the Shapiro–Wilk normality test and categorical data by Fisher’s exact test when cell counts <5 patients or chi-square test with Yate’s correction accordingly. Multivariable logistic regression models were constructed for LF and LS to identify independent risk factors based on univariate analysis results between high and low fibrosis and steatosis participants, respectively. Correlations between liver fibrosis and steatosis with several parameters were also explored by either point biserial or Pearson coefficient. Statistical analysis was performed in Python 3.6 and GraphPad 7.0a.

## Results

### Study Population and Female Subgroup Analysis

As shown in **Table 1**, a total of 52 patients with pSS (49 female patients, 94.2%) and 198 comparators (104 female patients, 52.5%) underwent TE. The median age (range) of pSS and comparators was 62.5 (30–81) and 55 (19–86) years, respectively. The median disease durations (range) of pSS from disease onset and diagnosis were 8 (1–46) and 3 (1–37) years, respectively. Comparators and pSS patients had comparable prevalence regarding T2DM, hyperlipidemia, and body mass index (BMI) (**Table 1**). The distribution of LS grading S0, S1, S2, and S3 was 22 (42.3%), 16 (30.8%), 6 (11.5%), and 8 (15.4%) in pSS patients, and 50 (20%), 63 (25.2%), 56 (22.4%), and 81 (32.4%) among comparators, respectively. The distribution of LF grading F0–F1, F2, and F3 was 50 (96.2%), 1 (1.9%), and 1 (1.9%) in pSS patients, and 214 (85.6%), 25 (10%), and 11 (4.4%) in comparators, respectively. After classification according to TE and clinical significance, high LS was found in 27% (*n* = 14) of pSS patients in comparison with 62% (*n* = 123) of the comparator group, while significant LF was found in 3.8% (*n* = 2) of SS patients and 17.2% (*n* = 37) in the comparator group.

**Table 1:** Comparison between patients with pSS and comparators.

	Sjögren’s Syndrome (*n* = 52)	Comparators (*n* = 198)	*p*-value
Age (years), median (range)	62.5 (30–81)	55 (19-86)	**0.0003**
Sex (female), *n* (%)	49 (94.2)	104 (52.5)	**<0.0001**
Body mass index, median (range)	27.1 (18.7-39.8)	28.1 (18.3-41.9)	0.1819
Diabetes mellitus, *n* (%)	12 (23.1)	50 (25.3)	0.8864
Hyperlipidemia, *n* (%)	23 (44.2)	109 (55.1)	0.2169
High liver steatosis (S ≥ 2), *n* (%)	14 (26.9)	123 (62.1)	**<0.0001**
Significant liver fibrosis (F ≥ 2), *n* (%)	2 (3.9)	34 (17.2)	**0.0136**

Bold p-values indicating statistically significant values.

Given the low number of male pSS patients in our cohort and the fact that pSS is a female predominant disease, the female pSS patients (*n* = 49) were compared with matched female comparators (*n* = 39) according to age by randomly selecting those female comparators with an age range that of the median age of female pSS ± 5 years. No difference was identified in terms of T2DM, hyperlipidemia, and BMI between the 2 groups, except for higher LS in the comparators (64% vs. 24%, *p* = 0.004) accompanied by a strong trend for LF (15.4% vs. 4%) but not reaching statistical significance (**Supplementary Table 1**). In this line, female pSS patients with low LS (*n* = 37) had no difference in terms of LF or other confounders compared to low LS comparators (*n* = 46), except for age (pSS = 64 vs. Comparators = 57 years, *p* = 0.023) (**Supplementary Table 2**).

### Association of Liver Fibrosis and Steatosis With pSS

From the 250 participants, 214 had insignificant and 36 significant LF, of whom 2 belonged to the study group and 34 belonged to the comparators. In univariable analyses including T2DM, hyperlipidemia, BMI, age, sex, steatosis, and disease status (pSS or comparators), significant LF was significantly associated with older age, higher LS, greater BMI, and disease status (comparators than pSS) (**Table 2**). In multivariable analysis, age was identified as the only independent risk factor for LF (**Table 3**; **Supplementary Figure 1**). In this line, LF was significantly correlated with age as suggested by point biserial analysis (*p* = 0.005), but with a weak coefficient (*r* = 0.18) (**Supplementary Figure 2**)

**Table 2:** Comparison between study participants with low and high liver fibrosis.

Characteristic	Univariable analysis			Multivariable analysis				
	Insignificant fibrosis (*n* = 214)	Advanced fibrosis (*n* = 36)	*p*-value	Regression coefficient	Odds ratio	Confidence interval low	Confidence interval upper	*p*-value
Age (years), median (range)	56	64	**0.0065**	0.057	1.059	1.019	1.100	**0.023**
Sex (female), *n* (%)	134 (62.6)	19 (52.8)	0.3492					
Body mass index, median (range)	27.6	29.7	**0.0083**	0.099	1.105	1.012	1.207	0.121
Diabetes mellitus, *n* (%)	48 (22.4)	14 (38.9)	0.0565					
Presence of Sjögren’s syndrome, *n* (%)	50 (23.4)	2 (5.6)	**0.0136**	−0.941	0.419	0.067	2.826	0.346
Hyperlipidemia, *n* (%)	113 (52.8)	19 (52.8)	0.8590					
High steatosis (S ≥ 2), *n* (%)	108 (50.5)	29 (80.6)	**0.0014**	0.344	1.473	0.355	6.167	0.620

Bold p-values indicating statistically significant values.

**Table 3:** Comparison between study participants with low and high steatosis.

Characteristic	Univariable analysis			Multivariable analysis				
	Low steatosis (*n* = 113)	High steatosis (*n* = 137)	*p*-value	Regression coefficient	Odds ratio	Confidence interval low	Confidence interval upper	*p*-value	
Age (years), median (range)	56	57	0.7278					
Sex (female), *n* (%)	83 (73.5)	70 (51.1)	**0.0005**	−4.819	0.6183	0.3259	1.173	0.144	
Body mass index, median (range)	25.7	29.3	**<0.0001**	2.372	1.268	1.2461	1.2902	**<0.001**	
Diabetes mellitus, *n* (%)	23 (20.4)	39 (28.5)	**<0.0001**					
Hyperlipidemia, *n* (%)	52 (46.0)	80 (58.4)	0.0682					
Presence of Sjögren’s syndrome, *n* (%)	38 (33.6)	14 (10.2)	**<0.0001**	−13.298	0.288	0.1263	0.6615	**0.0125**	

Bold p-values indicating statistically significant values.

From the 250 participants, 113 had low and 137 had high LS, of whom 14 belonged to the study group and 123 belonged to the comparators. In univariable analyses including age, sex, BMI, T2DM, hyperlipidemia, and disease status (pSS or comparators), high LS was significantly associated with sex, BMI, T2DM, and disease status (comparators than pSS) (**Table 3**). In multivariable analysis, BMI was positively correlated with high LS, while the presence of pSS was inversely associated with LS (**Table 2**; **Supplementary Figure 3**). Accordingly, LS was significantly correlated with BMI by point biserial analysis (*p* < 0.0001), but with a weak coefficient (*r* = 0.42) (**Supplementary Figure 4**)

### Clinical Characteristics of Patients With pSS According to Liver Fibrosis Status

Two pSS patients had significant LF; the first case referred to a 68-year-old female patient with a BMI of 29 kg/m^2^ and concomitant T2DM and arterial hypertension for 8 and 10 years, respectively, at the time of TE. She was diagnosed with pSS 8 years ago, complaining about persistent dry eyes and the laboratory workup revealed positive anti-nuclear antibodies (ANA) at a dilution of 1:160, positive anti-Ro/SSA autoantibodies, and a positive minor labial salivary gland biopsy. TE revealed stage 3 LS and stage 3 LF. She received proper treatment regarding her T2DM and arterial hypertension, and she never received methotrexate or prednisone for her primary disease. The second case referred to a 61-year-old female patient with a BMI of 33 kg/m^2^ at the time of TE. Her past medical history was otherwise unremarkable. She was diagnosed with pSS 11 years ago, based on dry mouth, dry eyes, Raynaud phenomenon, fatigue, and severe arthralgias of both hands. Laboratory tests were positive for ANA at a dilution of 1:160 exhibiting an anti-centromere antibody pattern at 1:1.280 while minor labial salivary biopsy was positive. She has been treated with methotrexate (cumulative dose of 7,800 mg) for a total of 11 years. TE revealed stage 2 LS and stage 2 LF.

Regarding SS patients with insignificant LF, the vast majority were female patients (47, 94%) with a median (range) age of 62.5 (30–81) years. The median disease duration (range) from diagnosis was 3 (1–37) years and that from pSS onset was 8 (1–46) years, while the median ESSDAI at disease diagnosis was 4 (0–20). The clinical manifestations of pSS patients with insignificant LF included oral dryness (88%, *n* = 44), ocular dryness (84%, *n* = 42), salivary gland enlargement (SGE) (22%, *n* = 11), arthritis (18%, *n* = 9), arthralgias (74%, *n* = 37), Raynaud’s phenomenon (31.8%, *n* = 19), interstitial lung involvement (10%, *n* = 5), small airways disease (6%, *n* = 3), interstitial renal disease (2% (*n* = 1), generalized lymphadenopathy (4%, *n* = 2), palpable purpura (14%, *n* = 14), and lymphoma (17%, *n* = 7); the autoantibody and laboratory profiles were as follows: anti-SSA antibodies (70%, *n* = 35), anti-SSB antibodies (58%, *n* = 29), rheumatoid factor (24%, *n* = 12), ANA antibodies (86%, *n* = 43), and low C4 (10%, *n* = 5). Sixteen of 50 patients (32%) were receiving corticosteroids at a median cumulative dose of 4,993 mg of prednisolone (range 610–94,800) for a median of 16.5 (range 8–272) months, and 9 patients (18%) were receiving methotrexate at a median cumulative dose of 3,130 mg (330–7,800) for a median of 63 (range 11–130) months. A total of 15 patients were receiving hydroxychloroquine at a median cumulative dose of 60 g (range 12–1,296 g) for a median of 10 (range 2–216) months. No correlations regarding treatment regimens were observed.

## Discussion

The fact that pSS affects liver frequently implies shared epitopes or epitope spreading across epithelial structures and particularly among salivary epithelium, biliary epithelium, and/or hepatocytes (27). In this case, a clinically silent chronic inflammatory process may evolve into liver parenchyma leading to LF (36–38). Fibrogenesis may be further promoted by several comorbidities or drugs, and therefore, it is reasonable to assume that LF might occur in higher prevalence among pSS patients compared to the general population. In the current study, LF and LS among pSS consecutive patients were assessed by TE, and a comparator group with individuals highly suspicious for NAFLD served as controls. The prevalence of significant LF among pSS reached 4% as opposed to 17% of the comparators, of whom 62% also showed a high degree of LS. The 2 groups also differed in terms of sex, age, and LS but not in other confounders of LF such as T2DM, hyperlipidemia, and BMI. However, in the multivariable analysis, only age and not LS was identified as an independent risk factor for LF, most likely due to the relatively low total number of individuals with significant LF. This particular association was further supported by the positive correlation between age and LF as attested through the point biserial analysis. Accordingly, subgroup analysis in female patients matched according to age between the 2 groups, and revealed a link between LF and LS as expected. Interestingly, in this specific cohort, high LS was positively associated with BMI and negatively associated with pSS; this negative association most likely represents a better control of pSS patients upon BMI or other comorbidities related to LS rather than an underlying protective pathogenetic mechanism. Taken together, it seems that pSS *per se* is not associated with significant LF or LS.

In our study, the prevalence of significant LF in pSS is similar to that of the general population (17). The estimated global prevalence of LF is approximately 5%, while NAFLD reaches 30% and thus pSS patients seem to display similar levels of LF and LS to the general population, most likely driven by classical risk factors (3, 6). In a previous study assessing the occurrence of LF in pSS patients, significant LF was found in 12% of cases after a disease duration period of 3.5 years, with low serum albumin, low white blood cell count, and high AST independently predicting significant LF (35). However, in that study, no comparator/control group was employed, and NAFLD/LS was not taken into account. At this point, we would like to emphasize that, since NAFLD is quite common in the general population, a control group of completely healthy individuals is not easy to be recruited. Furthermore, the reported association of significant LF with low white blood cell count could not be interpreted appropriately in the context of pSS (35). In comparison, pSS patients with insignificant LF in our cohort had similar disease duration, and were much older with more than a decade difference, and some of them received MTX and/or prednisone treatment, but still none developed clinically significant LF. On the contrary, the 2 pSS patients who presented with advanced LF had classical risk factors such as T2DM, high LS, or increased BMI, and one had been receiving MTX for many years, suggesting that pSS itself is most likely not associated with LF.

Our study has some limitations. Firstly, it is a retrospective study, since the TE was performed during the follow-up period and not at the time of pSS diagnosis; thus, it is by definition subject to selection and/or recall biases. Secondly, it is a single-center study on a fixed genetic background that may affect the outcome of both LF and LS. Thirdly, the number of recruited pSS patients and the disease duration may be relatively low—although the true disease course is longer—to study the fibrosis outcome of such a rare and long-standing process. Finally, the true effect of drugs cannot be easily evaluated, since apart from the cumulative dose and duration, specific drug combinations may interfere with the fibrogenesis procedure.

In conclusion, LF in pSS patients is most likely attributed to classical risk factors such as those in the general population and not to the disease *per se*, since pSS patients did not show an increased prevalence of LF compared to patients with NAFLD.

## Data Availability Statement

The raw data supporting the conclusions of this article will be made available by the authors, without undue reservation.

## Ethics Statement

Ethical review and approval was not required for the study on human participants in accordance with the local legislation and institutional requirements. The patients/participants provided their written informed consent to participate in this study.

## Author Contributions

TA, GP, AG, and AT were involved in study design. M-LK, A-DB, LC, and OA collected patients’ data and blood samples. TV performed all transient elastographies. Data analysis and interpretation were performed by VP, DF, and AG. All authors contributed to the article and approved the submitted version.

## Conflict of Interest

The authors declare that the research was conducted in the absence of any commercial or financial relationships that could be construed as a potential conflict of interest.

## Publisher’s Note

All claims expressed in this article are solely those of the authors and do not necessarily represent those of their affiliated organizations, or those of the publisher, the editors and the reviewers. Any product that may be evaluated in this article, or claim that may be made by its manufacturer, is not guaranteed or endorsed by the publisher.
